# Comprehensive analysis of integrin αvβ3/α6β1 in prognosis and immune escape of prostate cancer

**DOI:** 10.18632/aging.205131

**Published:** 2023-10-19

**Authors:** Yang Liu, Jia-Xin He, Bo Ji, Jin-Feng Wang, Lu Zhang, Zhong-Qi Pang, Jian-She Wang, Bei-Chen Ding, Ming-Hua Ren

**Affiliations:** 1 Department of Urinary Surgery, First Affiliated Hospital of Harbin Medical University, Harbin, Heilongjiang, China

**Keywords:** integrin αvβ3/α6β1, prostate cancer, prognosis, immune escape, risk model

## Abstract

Integrin αvβ3/α6β1 are crucial in the transduction of intercellular cancer information, while their roles in prostate cancer (PCa) remain poorly understood. Here, we systematically analyzed the transcriptome, single nucleotide polymorphisms (SNPs) and clinical data of 495 PCa patients from the TCGA database and verified them in 220 GEO patients, and qPCR was used to validate the expression of the model genes in our patients. First, we found that integrin αvβ3/α6β1 was negatively correlated with most immune cell infiltration and immune functions and closely associated with poor survival in TCGA patients. Then, we divided these patients into two groups according to the expression level of αvβ3/α6β1, intersected differentially expressed genes of the two groups with the GEO dataset and identified eight biochemical recurrence-related genes (BRGs), and these genes were verified by qPCR in our patients. Next, these BRGs were used to construct a prognostic risk model by applying LASSO Cox regression. We found that the high-risk (HR) group showed poorer OS, PFS, biochemical recurrence and clinical characteristics than the low-risk (LR) group. In addition, the HR group was mainly enriched in the cell cycle pathway and had a higher TP53 mutation rate than the LR group. More importantly, lower immune cell infiltration and immune function, higher expression of PD-L1, PD-1, and CTLA4, and higher immune exclusion scores were identified in the HR group, suggesting a higher possibility of immune escape. These findings suggested the key role of integrin αvβ3/α6β1 in predicting prognosis, TP53 mutation and immune escape in PCa.

## INTRODUCTION

Prostate cancer (PCa), a common malignant cancer, is still one of the top ten cancers in the world, is the most commonly diagnosed cancer in the USA and is ranked sixth in morbidity and seventh in mortality in China [1, 2]. At present, PCa therapies include surgery, androgen-deprivation therapy (ADT), radiation therapy (RT), ablative therapy, chemotherapy, and immune check-point immunotherapy [3]. Although many early clinical PCa patients with low-risk or intermediate-risk disease could be greatly cured by performing surgery, ablative therapies, or RT, they might possibly develop biochemical recurrence of PCa [4, 5]. In addition, more advanced PCa patients with high risk who received ADT and chemotherapy finally progressed to refractory castration-resistant prostate cancer (CRPC), which was considered to be closely associated with the poor clinical prognostic factor androgen receptor (AR) in PCa [3, 6]. Immune checkpoint inhibitor (ICI) therapy, as a newly developed treatment, seems to be an efficient method to relieve CRPC [7]. Unfortunately, it was reported that some immune therapies targeting T-cell immune checkpoints, including CTLA-4, PD-L1 and PD-1, have not been demonstrated to be significantly efficient in PCa, which might be connected with the poor tumor immune microenvironment in PCa [8]. According to a literature review, PCa is considered a “cold” tumor with low levels of infiltrating T cells, defective function of antigen presenting cells (APCs) and high levels of infiltrating immunosuppressive cells such as MDSCs and Tregs, the latter of which can secrete cytokines to suppress immune function and inhibit immune cell activation in the tumor microenvironment (TME), contributing to the immune escape of PCa cells [9]. Therefore, new methods are needed to accurately predict the prognosis and immune escape of PCa.

Integrins, known as a kind of transmembrane glycoprotein, are noncovalently composed of two different heterodimers composed of 18 α and 8 β integrin polypeptides, which interact with various ligands to produce many pathophysiological effects, including cell adhesion, inflammation, and neoplasm [10]. Among others, integrins αvβ3 and α6β1 were proven to be overexpressed in PCa patients and were connected with tumor growth, angiogenesis and metastasis in PCa [11]. Rubenstein, C. S. et al. demonstrated that PCa cells expressing α6 integrin (DU145 α6 WT) produced a 3D invasive network on laminin-containing Matrigel and invaded smooth muscle both *in vitro* and *in vivo*, and integrin α6 enhanced the intercellular biophysical properties in PCa [12]. Otherwise, integrin αvβ3 is regarded as a potential biomarker for the early detection of neuroendocrine PCa, and according to the literature, integrin β1 is strongly connected with PCa recurrence after radical surgery, suggesting that integrins play a crucial role in the invasion, progression and prognosis of PCa [13, 14]. Furthermore, integrins and integrin ligands also play vital roles in the infiltration and activation of T cells and the tumor immune microenvironment, which shows a dual effect of promotion and antitumor activity in cancers [15]. Some reviews have reported the potential association between abnormally expressed integrins and PCa, nevertheless, a comprehensive analysis of the value of integrin αvβ3/α6β1 in predicting prognosis and immune escape in PCa is still inadequate [16].

In this work, we intend to construct a risk model based on integrin αvβ3/α6β1 to effectively predict prognosis and immune escape in PCa, and these findings may also provide new potential targets for the precision treatment of PCa.

## RESULTS

### Integrin αvβ3/α6β1 were closely associated with poor immunity in PCa patients

**Figure 1:**
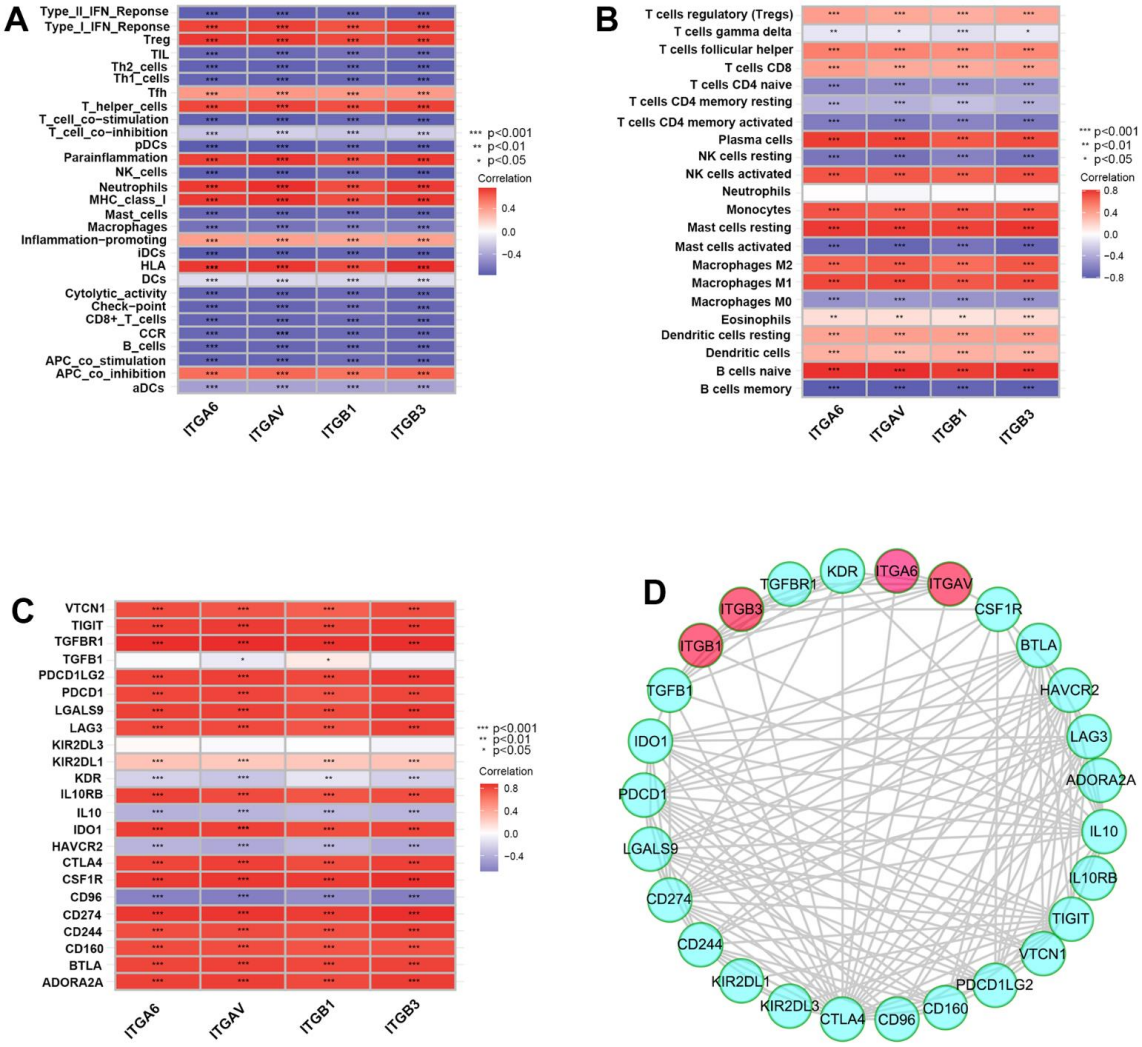
**Integrin αvβ3/α6β1 were closely correlated with poor immunity in patients with prostate cancer (PCa).** (**A**–**C**) Correlation plots of integrin αvβ3/α6β1 with immune functions (**A**), immune cell infiltration (**B**) and immunosuppressive genes (**C**) in PCa patients. (**D**) The protein-protein interaction network of integrin αvβ3/α6β1 and immunosuppressive genes.

First, we systematically analyzed the correlations among integrin αvβ3/α6β1 with immune cell infiltration, immune functions and immunosuppressive genes. Interestingly, we found that integrin αvβ3/α6β1 was negatively associated with immune functions, including APC co-stimulation, cytolytic activity, T-cell co-stimulation and the type II IFN response, and integrin αvβ3/α6β1 was negatively correlated with most immune functional cells, including DCs, B cells, NK cells, CD8^+^ T cells, mast cells, Th cells, macrophages, and other tumor infiltrating lymphocytes (TILs) (Figure 1A). In contrast, integrin αvβ3/α6β1 was positively correlated with negative immune function, including APC co-inhibition and Tregs. Moreover, integrin αvβ3/α6β1 was negatively correlated with memory B cells, activated CD4+ memory T cells and activated mast cells, which could reduce immune cellular functions in the tumor microenvironment (TME) of PCa patients (Figure 1B). Low infiltration of immune cells in the TME and immune cell dysfunction may contribute to the immune escape of PCa cells. In addition, it also showed close positive correlations when intersecting integrin αvβ3/α6β1 with some classical immunosuppressive genes that were connected with immune escape, such as CTLA4, IL10RB, PDCD1LG2 (B7-DC), CD274 (B7-H1), TGFBR1, and PDCD1 (Figure 1C). Similarly, the protein-protein interaction network (PPI) showed tight correlations among these genes with integrin αvβ3/α6β1, which indicated mutually regulated relationships between integrin αvβ3/α6β1 and those immunosuppressive genes or their expressed proteins (Figure 1D). Together, these findings preliminarily suggested that integrin αvβ3/α6β1 might be strongly correlated with poor immunity and immune escape in PCa.

### Integrin αvβ3/α6β1 were closely associated with poor prognosis of PCa patients

**Figure 2:**
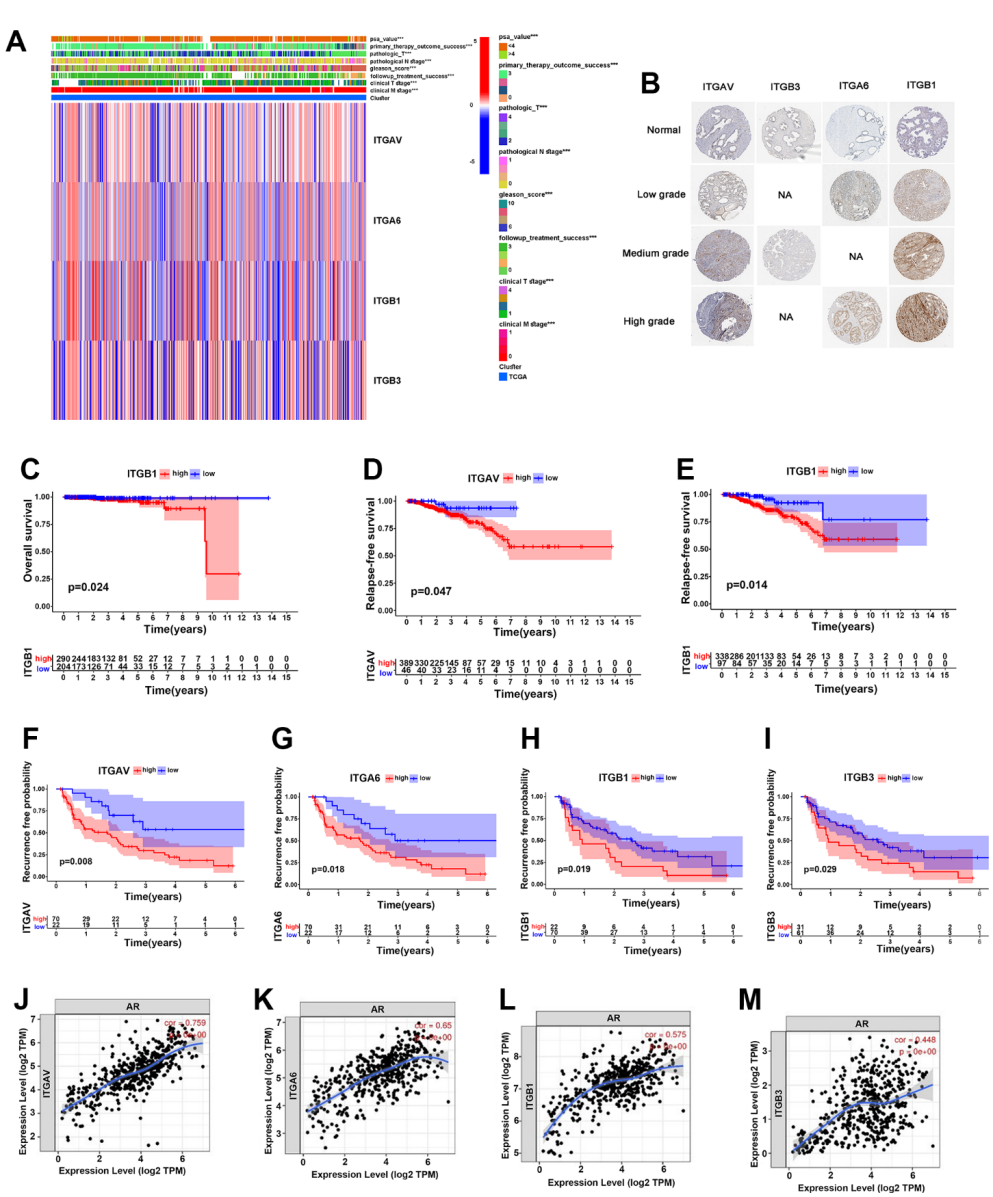
**Integrin αvβ3/α6β1 were closely correlated with poor prognosis of PCa patients.** (**A**) Heatmap of integrin αvβ3/α6β1 expression and clinical characteristics of 496 PCa patients in the TCGA cohort. (**B**) The immunohistochemistry of integrin αvβ3/α6β1 between normal prostate tissues and different grades of PCa tissues in the Human Protein Atlas (HPA). (**C**) Kaplan-Meier analysis of the OS of patients with high and low ITGB1 expression in the TCGA cohort. (**D**, **E**) Kaplan-Meier analysis of the RFS of patients with high and low ITGAV (**D**) and ITGB1 (**E**) expression in the TCGA cohort. (**F**–**I**) Kaplan-Meier analysis of the biochemical recurrence of patients with high and low ITGAV, ITGA6, ITGB1, and ITGB3 expression in the TCGA cohort. (**J**–**M**) Correlations between ITGAV, ITGA6, ITGB1, ITGB3 and androgen receptor (AR).

In our study, a heatmap was established to clearly observe the expression levels of integrin αvβ3/α6β1 and clinical characteristics, including PSA value, primary therapy outcome success, pathological T and N stages, Gleason score, treatment success and clinical T and N stages, of 496 PCa patients in the TCGA cohort, and the results implied that integrins ITGAV, ITGA6, ITGB1 and ITGB3 were closely associated with these clinical features (Figure 2A). Additionally, we found higher expression levels of these integrins in low-grade PCa, especially in medium-grade and high-grade PCa, than in normal prostate tissues, according to the immunohistochemical results from the Human Protein Atlas (HPA) (Figure 2B). To explore the effects of integrin αvβ3/α6β1 on the prognosis of PCa patients, we performed Kaplan-Meier analysis for overall survival (OS), relapse-free survival (RFS) and biochemical recurrence based on the expression levels of integrin ITGAV, ITGA6, ITGB1 and ITGB3 in these samples. The results showed that the OS for the samples with high expression levels of ITGB1 was significantly lower than that for the samples with low expression levels of ITGB1 (Figure 2C). Likewise, the RFS for the samples with high expression levels of ITGAV and ITGB1 was markedly lower than that for the samples with low expression levels of ITGAV and ITGB1, which might indicate that integrin αvβ3/α6β1 could be a potential prognostic element in PCa (Figure 2D, 2E). Furthermore, PCa patients who expressed high levels of integrin ITGAV, ITGA6, ITGB1 and ITGB3 had a higher proportion of biochemical recurrence than patients with low expression levels of integrin αvβ3/α6β1 (Figure 2F–2I). Moreover, we further investigated the correlations among the expression levels of the integrins ITGAV, ITGA6, ITGB1 and ITGB3 and the expression levels of AR in PCa patients. According to literature reports, androgen receptor (AR) is one of the prognostic factors in PCa, and contributes to the progression and recurrence of PCa. Surprisingly, there were strong correlations among the integrins ITGAV, ITGA6, ITGB1 and ITGB3 with AR in PCa patients, whose expression levels for cor-values were 0.759, 0.650, 0.575, and 0.448, respectively (Figure 2J–2M). In summary, these results suggested that integrin αvβ3/α6β1 could be a negative prognostic factor for PCa.

### A prognostic risk model based on integrin αvβ3/α6β1 for PCa patients

**Figure 3:**
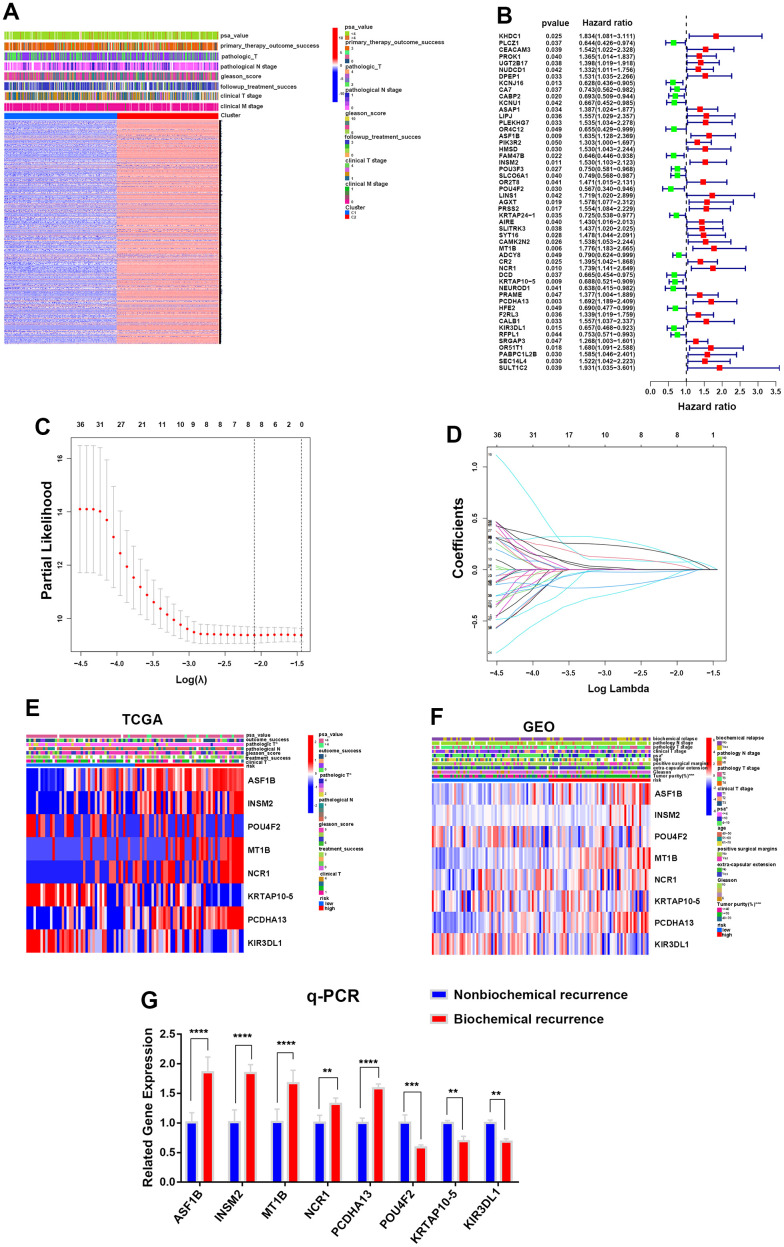
**Construction of a prognostic risk model for PCa according to integrin αvβ3/α6β1.** (**A**) Heatmap of differentially expressed genes (DEGs) and clinical characteristics for two clusters in the TCGA cohort. (**B**) Forest plot of 51 DEGs interfering with the biochemical recurrence of PCa in the TCGA cohort. (**C**) LASSO Cox regression analysis for eight biochemical recurrence-related genes in both the TCGA and GEO cohorts. (**D**) LASSO Cox regression of cross-validation between the TCGA and GEO cohorts. (**E**, **F**) Heatmaps of the expression of 8 biochemical recurrence-related genes in the TCGA and GEO cohorts. (**G**) qPCR was used to verify the gene expression levels of the constructed model in biochemically recurrent and nonbiochemically recurrent PCa.

First, we divided the PCa patients into two clusters (C1 and C2) according to the average expression levels of whole integrin αvβ3/α6β1 (ITGAV, ITGA6, ITGB1, ITGB3) in the TCGA cohort, and we performed gene cluster analysis for the two clusters to identify significant DEGs based on the whole genetic transcriptomes of 496 PCa patients in the TCGA dataset, and 13994 DEGs were identified in the two clusters (Figure 3A). In addition, information on the clinical characteristics of these PCa patients was also manifested in the heatmap. Next, 51 DEGs were identified as prominently affecting the biochemical recurrence of PCa by conducting univariate Cox analysis, including 33 DEGs (hazard ratio >1) that increased the risk of biochemical recurrence and 18 DEGs (hazard ratio <1) that decreased this risk in the TCGA cohort (Figure 3B). Subsequently, we intersected these 51 DEGs with the DEGs acquired from the GEO dataset and successfully identified eight biochemical recurrence-related genes (ASF1B, INSM2, POU4F2, MT1B, NCR1, KRTAP10-5, PCDHA13 and KIR3DL1) in both the TCGA and GEO cohorts. Next, we constructed a risk model using the optimum γ value based on the eight genes by applying LASSO Cox regression analysis (Figure 3C, 3D). In addition, we calculated the risk score of these 8 biochemical recurrence-related genes by running the formula: Risk score= (0.0741808537008443 * expression of ASF1B) + (0.0584797685631388 * expression of INSM2) + (-0.0665901093358763 * expression of POU4F2) + (0.152621619239078 * expression of MT1B) + (0.00167204457436465 * expression of NCR1) + (-0.0181031499555924 * expression of KRTAP10-5) + (0.151620509953615 * expression of PCDHA13) + (-0.0816400681584183 * expression of KIR3DL1).

Based on the median risk score, PCa patients in the TCGA cohort and GEO cohort, which had complete biochemical recurrence information, were objectively divided into the HR group and LR group (Supplementary Figure 1A). The genes ASF1B, INSM2, MT1B, NCR1 and PCDHA13 were highly expressed in the HR group, while the genes POU4F2, KRTAP10-5 and KIR3DL1 were highly expressed in the LR group in both the TCGA and GEO cohorts (Figure 3E, 3F). To further confirm the relationship between these genes and biochemical recurrence, we selected 5 cases of biochemically recurrent prostate cancer and 5 cases of nonbiochemically recurrent prostate cancer for RNA extraction. The results showed that the expression levels of ASF1B, INSM2, MT1B, NCR1 and PCDHA13 in biochemically recurrent PCa were higher than those in nonbiochemically recurrent PCa, while POU4F2, KRTAP10-5 and KIR3DL1 were significantly lower in biochemically recurrent PCa than in nonbiochemically recurrent PCa (Figure 3G). Additionally, we were able to find that there were more dead samples that were regarded as the HR group located in the area of higher risk scores with increasing risk scores of patients. In contrast, the other survivors who were regarded as the LR group were more likely to be located in areas with lower risk scores, and they had longer survival times than the HR group (Supplementary Figure 1B). Otherwise, we applied principal component analysis (PCA) and t-distributed stochastic neighbor embedding (t-SNE) analysis to demonstrate that samples in the HR and LR groups could be greatly separated (Supplementary Figure 1C, 1D).

**Figure 4:**
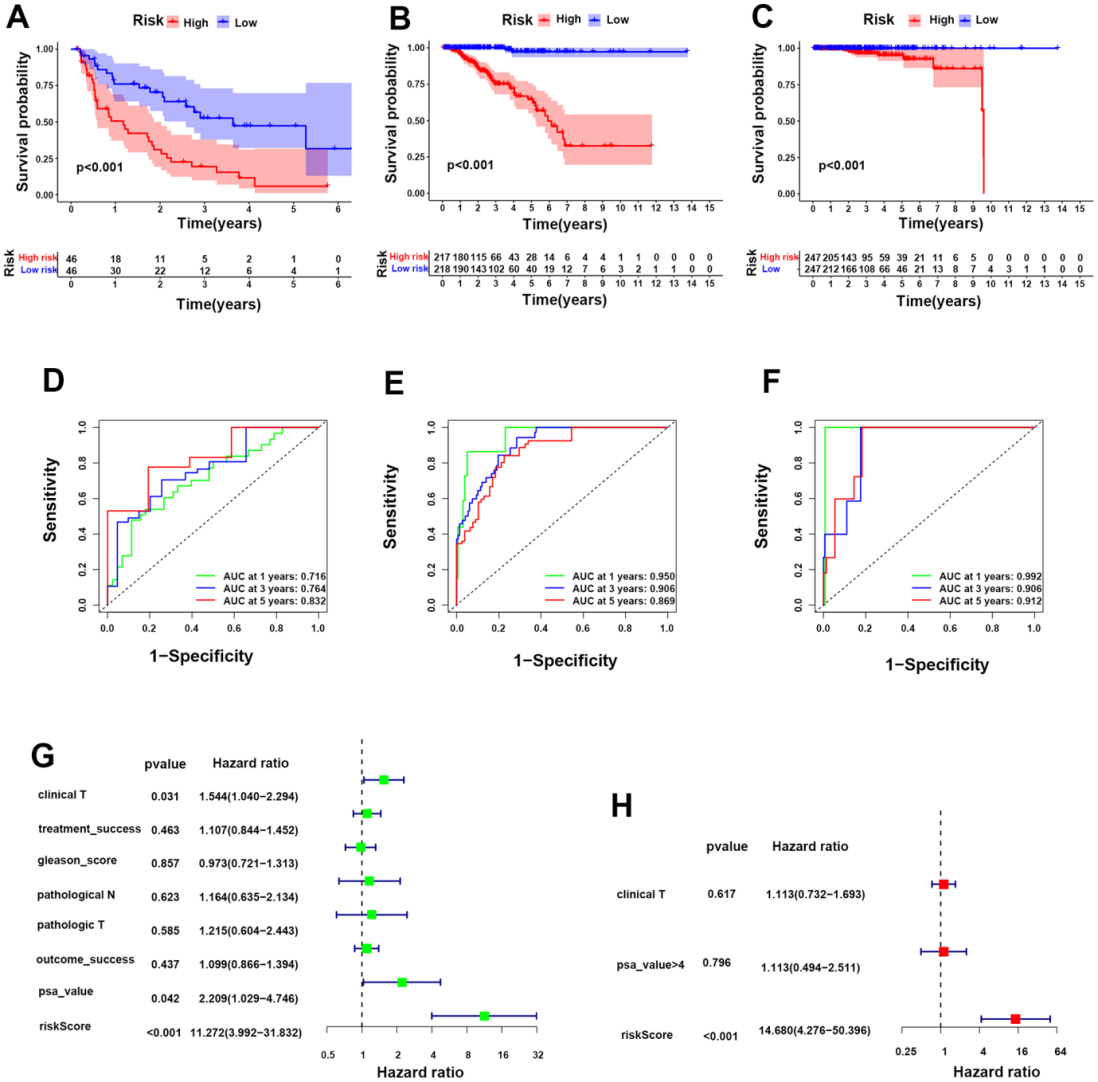
**Construction of a prognostic risk model for PCa according to integrin αvβ3/α6β1.** (**A**–**C**) Kaplan-Meier analysis for biochemical recurrence, PFS and OS in the two risk groups. (**D**–**F**) ROC analysis was performed to validate the predictive power for biochemical recurrence, PFS and OS of PCa patients in our model. (**G, H**) Forest maps of univariate and multivariate analyses for risk score in our model.

Furthermore, we carried out Kaplan-Meier analysis for biochemical recurrence between the two groups, and the results revealed worse biochemical recurrence probabilities for PCa patients in the HR group than in the LR group (Figure 4A). ROC analysis was performed to prove the predictive ability of our model for biochemical recurrence, and the area under the ROC curve (AUC) was 0.716, 0.764, and 0.832 for 1, 3, and 5 years, respectively (Figure 4D). Likewise, worse progression-free survival (PFS) and OS were observed in the HR group than in the LR group (Figure 4B, 4C). The AUC values for PFS were 0.950 at 1 year, 0.906 at 3 years, and 0.869 at 5 years, while the AUC values for OS were 0.992 at 1 year, 0.906 at 3 years, and 0.912 at 5 years (Figure 4E, 4F). Moreover, univariate and multivariate analyses showed that the risk score was an independent risk factor for PCa patients (Figure 4G, 4H). These evidences illustrate that our risk model was powerful for predicting the prognosis of PCa patients.

### The HR risk group predicted poor clinical features of PCa patients

**Figure 5:**
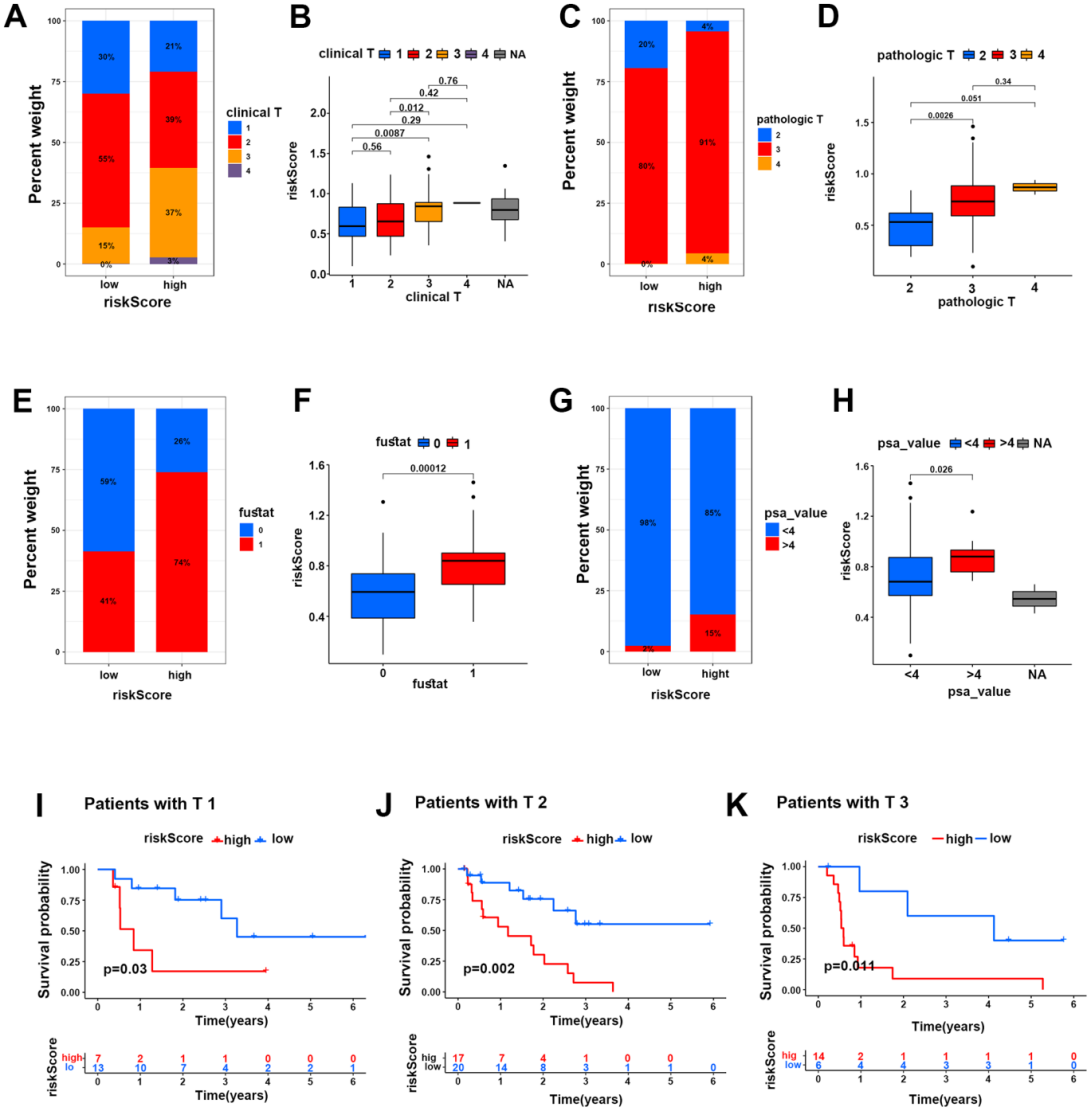
**The HR risk group predicted poor clinical characteristics of PCa patients.** (**A**–**H**) Percent weights and risk scores of clinical T grades (**A**, **B**), pathological T grades (**C**, **D**), fustat (**E**, **F**) and PSA values (**G**, **H**) of the two risk groups in the TCGA cohort. (**I**–**K**) Kaplan-Meier survival analysis for PCa patients with clinical T1, T2 and T3 grades in two risk groups in the TCGA cohort.

On the other hand, we investigated the correlations of the two risk groups with clinical features in PCa patients. The results showed that the percent weight of clinical and pathologic T3-stage and T4-stage in the HR group was higher than that in the LR group (40% vs 15% and 95% vs 80%), and the risk score of clinical and pathologic T3-stage and T4-stage was higher than that of clinical and pathologic T1-stage and T2-stage, respectively (Figure 5A–5D), suggesting that more patients suffered advanced PCa in the HR group. In addition, the percent weight of fustat-1 (the dead patients) in the HR group was markedly higher than that in the LR group (74% vs 41%), which was the same result as the risk score in the two groups (Figure 5E, 5F). In addition, the percent weight of PSA (value >4), which could greatly identify PCa in patients, was dramatically higher in the HR group than in the LR group (15% vs 2%), and the risk score of PSA (value >4) was higher than that of PSA (value <4) in the two groups as well (Figure 5G, 5H).

Moreover, Kaplan-Meier survival analysis was performed for the two risk groups under subgroups of clinical characteristics of PCa patients. The results showed that PCa patients with clinical T1-stage, T2-stage and T3-stage disease probably suffered more mortality in the HR group than in the LR group (Figure 5I–5K). Similarly, the patients with Gleason score 7-9, PSA (value >4 and <4), pathologic stages of T2-T3 and N0-N1 both suffered lower survival probability in the HR group than in the LR group (Supplement Figure 3A–3I). This evidence strongly demonstrated that the HR group could well predict poor survival in PCa patients.

### Genetic mutation status and functional enrichment analysis for the two risk groups

**Figure 6:**
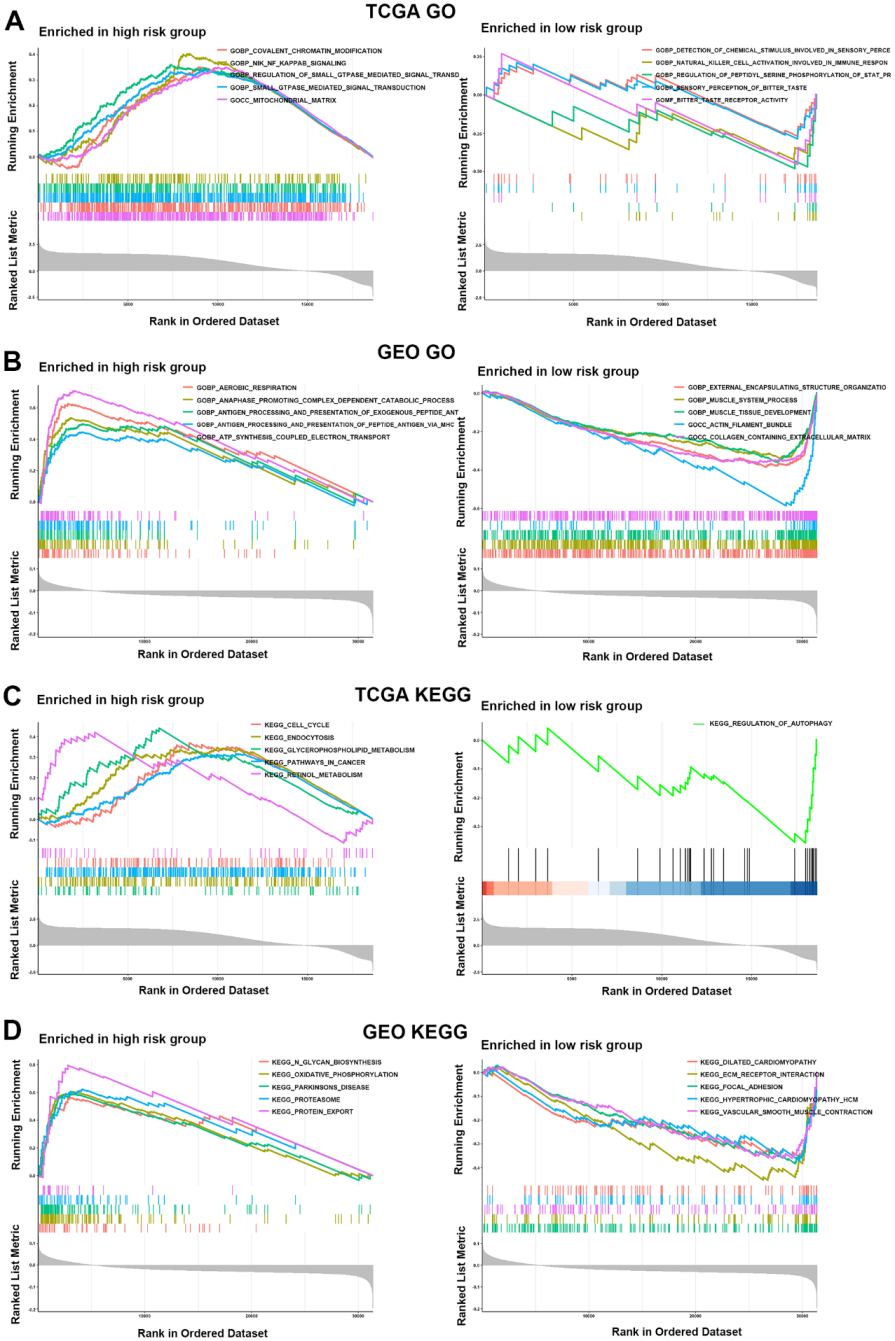
**Functional enrichment analysis for the two risk groups.** (**A**, **B**) GO enrichment analysis for two risk groups in the TCGA (**A**) and GEO (**B**) cohorts. (**C**, **D**) KEGG enrichment analysis in the HR and LR groups in the TCGA (**C**) and GEO (**D**) cohorts.

In the TCGA cohort, the GO enrichment analysis showed that GOBP: NIK (NF-κB induced kinase) NF-κB signaling, GOBP: ras protein signal transduction, GOBP: canonical Wnt signaling pathway and GOBP: positive regulation of interleukin-10 production was markedly enriched in the HR group compared with the LR group. Moreover, according to the GEO cohort, GOBP: transforming growth factor beta1 production and GOMF: heat shock protein binding was more enriched in the HR group than in the LR group. Interleukin-1-mediated signaling and FC receptor signaling were more significantly enriched in the HR group than in the LR group in both the TCGA and GEO cohorts (Figure 6A, 6B and Supplementary Tables 1, 2). In addition, KEGG enrichment analysis showed that KEGG: T-cell and B-cell receptor signaling pathway, KEGG: P53 signaling pathway, KEGG: wnt signaling pathway, TGF beta signaling pathway, chemokine signaling pathway, KEGG: cell cycle and KEGG: MAPK signaling pathway were highly enriched in the HR group compared with the LR group in the TCGA or GEO cohorts (Figure 6C, 6D and Supplementary Tables 3, 4). Based on these results, we can note that most of the functions or pathways enriched in the HR group are associated with poor patient outcomes. Therefore, these evidences might reveal the potential mechanisms for poor prognosis in the HR group.

**Figure 7:**
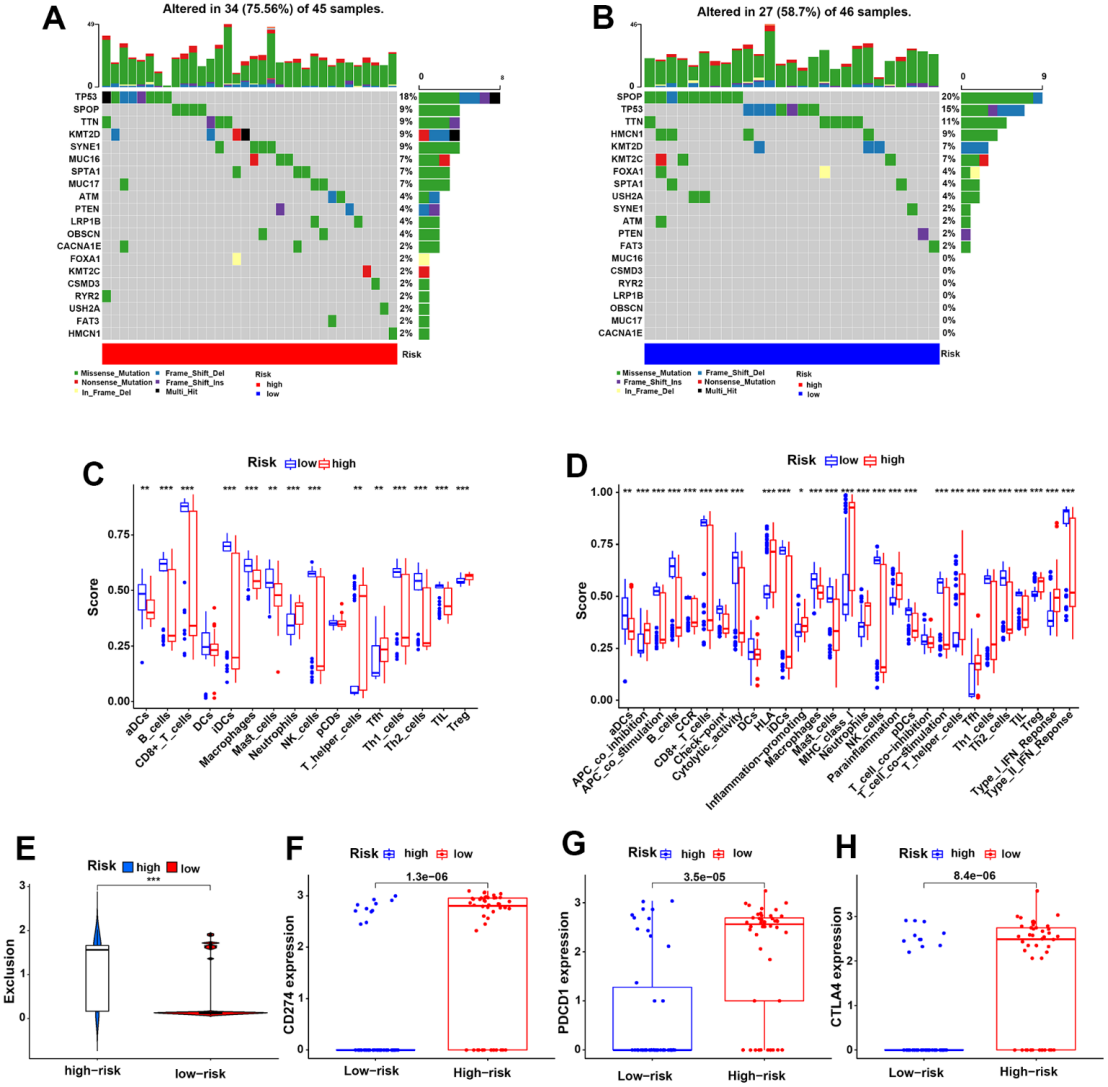
**Genetic mutation status and the HR group predicted a higher risk of immune evasion in PCa patients.** (**A**, **B**) The 20 most frequently mutated genes in the HR and LR groups in the TCGA cohort. (**C**, **D**) The discrepancies in 16 infiltrating immune cells and 29 immune functions of PCa patients in the two risk groups in the TCGA cohort. (**E**) The different risks of immune exclusion of PCa patients between the HR and LR groups in the TCGA cohort. (**F**–**H**) The expression differences in the immune checkpoints CD274 (**F**), PDCD1 (**G**) and CTLA-4 (**H**) for the two risk groups in the TCGA cohort.

To evaluate the difference in genetic mutation status in the two risk groups, we used Maftools to identify the whole gene mutation in 91 PCa patients with complete biochemical recurrence data from the TCGA dataset, of which the 20 most frequently mutated genes are shown in Figure 7A, 7B. We noticed a higher frequently mutated ratio in the HR group than in the LR group (75.56% vs 58.70%). Moreover, the TP53 and KMT2D genes were more often mutated in the HR group than in the LR group (18% vs 15% and 9% vs 7%). However, the gene SPOP mutation rates were higher in the LR group than in the HR group (20% vs 9%), which could be attributed to genetic heterogeneity. Collectively, these mutated genes might partially contribute to the poor clinical prognosis of PCa in the HR group.

### The HR group predicted a higher risk of immune escape for PCa patients

Moreover, we further investigated the components of immune cells that infiltrated the TME of PCa between the two groups in TCGA samples (Figure 7C). The results showed lower infiltration of DCs, B cells, CD8^+^ T cells, macrophages, NK cells, Th cells and TILs in the HR group than in the LR group. In contrast, negatively regulated immune cells, such as Treg cells, were higher in the HR group than in the LR group. To further investigate the influences of immune cells on our patients, Kaplan-Meier analysis was performed for PFS, OS and biochemical recurrence in the TCGA cohort. As expected, the high expression levels of CD8^+^ T cells, NK cells and macrophages exhibited better PFS, OS or biochemical recurrence, while the high expression levels of Tregs exhibited poor PFS and OS (Supplementary Figure 2A–2H). These results were consistent with conventional wisdom.

Additionally, immune functions were also investigated in the HR and LR groups, and the results showed that poor immune cell functions, including aDCs, B cells, CD8+ T cells, NK cells and macrophages, and better Treg cell functions were found in the HR group than in the LR group. Immune cell functions such as APC co-stimulation, T-cell co-stimulation and the type II IFN response were also lower in the HR group than in the LR group (Figure 7D). These evidences illustrate worse immune functions in the HR group, which might contribute to tumor immune escape in PCa. Next, we compared the expression of immune checkpoints in the risk groups and found that the HR group had higher expression of PD-1 (CD274), PD-L1 (PDCD1), and CTLA-4 than the LR group, which might lead to a higher risk of immune escape in PCa (Figure 7F–7H). In addition, we scored the risk of immune exclusion in our patients; surprisingly, we found that the immune exclusion score was higher in the HR group than in the LR group (Figure 7E). These findings suggested that the HR risk group might suffer a higher risk of immune escape in PCa patients.

## DISCUSSION

Integrins are ubiquitous heterodimeric transmembrane glycoprotein receptors that interact with ligands in cells and the extracellular matrix and mainly act as bidirectional signaling proteins to regulate various physiological functions of cells, such as mediating cell adhesion and promoting tumorigenesis, progression, and metastasis in tumors [17, 18]. The biological functions of integrin αvβ3 and integrinα6β1 are particularly important and have been extensively studied. Integrin αvβ3 is necessary for tumor cell adhesion to the extracellular matrix (ECM) by targeting RGD (Arg-Gly-Asp) in fibronectin and regulates MMP (especially MMP-2 and MMP-9) expression through the PI3K signaling pathway to hydrolyze collagen in the ECM [19]. It has been reported that integrin αvβ3 participates in almost the entire growth processes of PCa, especially contributing to progression, angiogenesis and metastasis by combining with ligands expressing the RGD sequence, for instance, bone sialoprotein (BSP) and osteopontin (OPN) in PCa [19]. Kim B et al. confirmed that integrin αvβ3 upregulated CCN2 (a connective tissue growth factor) to activate downstream signaling, contributing to the aggressiveness of PCa bone metastasis [20]. Likewise, chemokine C-C motif ligand 2 (CCL2) was proven to increase the expression of integrin αvβ3, which enhanced the migration of PCa [21]. In addition, a recent study proposed that integrin α6β1 promoted PCa cells to invade surrounding nerves and supported PCa bone metastasis when laminin was combined with integrin α6β1 [22]. The upregulated expression of integrin α6β1, which was stimulated by active androgen receptor (AR) in castration-resistant prostate cancer (CRPC), could promote the accumulation of Bnip3 to prevent the apoptosis of CRPC cells [23]. Moreover, our previous study revealed that endostatin 33 peptide is a disintegrin α6β1 agent that exerts antitumor activity by inhibiting the PI3K-Akt signaling pathway in prostate cancer [24]. In this work, we integrated the genetic characteristics of integrins αvβ3 and α6β1, focused more on the derivative value of integrin αvβ3/α6β1 rather than the gene itself, constructed a risk model based on integrin αvβ3/α6β1 and revealed its critical value in predicting OS, PFS, and biochemical recurrence in PCa patients. This evidence indicates that the risk model based on integrin αvβ3/α6β1 may be an indicator of the diagnosis and prognosis of PCa and that the genes that construct the model might be new therapeutic targets.

On the other hand, several studies have suggested a close relationship between integrins and tumor immunity. Bagati A et al. confirmed that breast cancer could escape CD8^+^ T-cell attack through the integrin αvβ6-TGFβ-SOX4 pathway, which significantly decreases tumor sensitivity to CD8^+^ T cells [25]. According to a literature review, integrin αv especially upregulates TGF beta, contributing to promoting angiogenesis and immune suppression in cancers [26]. Integrin αvβ3 was also proven to promote immune escape by regulating the interferon signaling pathway and increasing PD-L1 expression in cancers [19, 27]. Additionally, Yang H et al. showed that ITGB1 suppressed T-cell function, contributing to immune evasion and a low response to immune checkpoint treatment in pancreatic cancer [28]. Despite this, the effect of integrins on immune function in PCa is not well understood. In our work, we found that integrin αvβ3/α6β1 were negatively correlated with most immune cell infiltration and immune functions in PCa patients, suggesting that integrin αvβ3/α6β1 might be a reason for immune evasion of PCa, which is worth further discussion in follow-up studies.

In our study, we identified eight biochemical recurrence-related genes (ASF1B, INSM2, POU4F2, MT1B, NCR1, KRTAP10-5, PCDHA13, and KIR3DL1) to establish a risk model. The expression of ASF1B, INSM2, MT1B, and PCDHA13 was markedly upregulated in the HR group. ASF1B (antisilencing function 1B) is a histone H3-H4 chaperone protein that regulates the functions of chromatin in cells [29]. Han G et al. demonstrated that ASF1B was overexpressed in PCa tissues, which was closely connected with the low OS of PCa patients, since ASF1B could promote tumorigenesis through the ASF1B-PI3K\AKT signaling pathway mediating the cell cycle in PCa [30]. Similarly, it was reported that ASF1B was significantly correlated with poor prognosis in several other cancer patients [31, 32]. Cao H et al. demonstrated that the upregulated expression of INSM2 promoted tumorigenesis and progression by regulating lipid metabolism in neuroblastoma and led to poor prognosis in neuroblastoma patients [33]. MT1B, known as an important isoform of metallothioneins (MTs), was reported to participate in the regulation of copper-zinc homeostasis, and MTs play an important role in tumorigenesis, angiogenesis, metastasis, proliferation and immunomodulation of cancers [34]. Wang KH et al. illustrated that methylated PCDHA13 and PCDHA4 were closely associated with the severity and progression of cervical carcinoma, and PCDHA13 and PCDHA3 were considered screening biomarkers with more specificity and equal sensitivity when combined with HPV to test cervical cancers compared with the HPV test alone [35]. Together, these evidences show that the overexpressed genes in the HR group were strongly associated with the poor prognosis of cancers, which supported our results that the HR group could predict the prognosis of PCa in our model.

As recently described, immune checkpoint inhibitors (ICIs), including CTLA4 inhibitors and PD1 and PD-L1 inhibitors, have been approved for treating many cancers, such as kidney cancer, melanoma, urothelial cancer and lung cancer, and have achieved good outcomes [36]. However, according to the literature reviews, the monotherapy and combination of ICIs showed limited benefits with low levels of therapeutic responses in CRPC, without a prominent survival improvement, while the combination therapies of ICIs with vaccines, androgen receptor targeting inhibitors (ARTi), chemotherapeutic drugs, poly ADP ribose polymerase (PARP) inhibitors or tyrosine kinase inhibitors (TKI) exhibited a prospective method for the treatment of CRPC [37–39]. Interestingly, Dong M et al. proved that the complex consisting of CUL3 and SPOP proteins could lead to the degradation of PD-L1 through ubiquitination, inhibiting the immune evasion of ovarian cancers [40]. This evidence strongly indicates the importance of seeking new immunotherapy targets in cancer treatment. More importantly, regulating the integrin pathway and expression might enhance the antitumor effects of ICIs in cancers, and newly developed antibodies aimed at integrins are worth evaluating in PCa in the future [41, 42]. In our study, we developed an integrin αvβ3/α6β1-based risk model that can accurately predict immune cell infiltration, immune cell function, immune exclusion score and immune checkpoint levels (PD-1, PD-L1 and CTLA-4) in PCa patients. These results indicate that integrin αvβ3/α6β1 and the constructed genes in our model are expected to be novel molecular targets for immune escape therapy of PCa.

In conclusion, we established a prognostic risk model based on integrin αvβ3/α6β1, which revealed the important role of integrin αvβ3/α6β1 in the prognosis, TP53 mutation and immune escape of PCa. These evidences might provide new medical therapeutic targets for PCa patients.

## MATERIALS AND METHODS

### Data sources

The data of genetic transcriptomes, tumor mutational burden (TMB), SNPs and clinical characteristics for 495 PCa samples were acquired from the TCGA dataset (https://tcga-data.nci.nih.gov/tcga/), and the genetic transcriptomes and clinical features for 220 PCa cases were downloaded from the GEO dataset (http://www.ncbi.nlm.nih.gov/geo/). The results of immunohistochemistry (IHC) of prostate tissues and PCa were acquired from the Human Protein Atlas (HPA) dataset (https://www.proteinatlas.org/). The expression level of integrin αvβ3/α6β1 in each clinical PCa patient was determined by the mean of the expression levels of the integrin subunits ITGAV, ITGA6, ITGB1, and ITGB3.

### Immune correlation analysis and protein-protein interaction network

We utilized CIBERSORT to acquire information on immune infiltrating cells and immune functions in PCa patients. Pearson correlation analysis was performed for integrin αvβ3/α6β1, immune cells, immune functions and immunosuppressive genes in PCa patients. In addition, the protein-protein interaction network (PPI) for integrin αvβ3/α6β1 with immunosuppressive genes was formed by applying STRING (https://string-db.org/) and Cytoscape software.

### Identification of differentially expressed genes

First, the gene expression data in both the TCGA and GEO cohorts were standardized with the formula log2(x+1), and we intersected the TCGA genetic matrix with the GEO genetic matrix to acquire the expression data of the same genes in both the TCGA and GEO cohorts. Then, the data of differentially expressed genes (DEGs) were finally identified by applying the edge R package with FDR < 0.05 and |log2FC| ≥ 0.585 in both TCGA and GEO cohorts.

### Establishment of the prognostic risk model

We intersected biochemical recurrence-related genes in both the TCGA and GEO databases, and eight biochemical recurrence-related genes were finally identified, which were used to establish our prognostic risk model by applying LASSO Cox regression analysis. In our work, the risk score was calculated by $\sum_{\text{i}}^{8}\text{Ai}\times \text{Bi}$ (A: coefficients, B: gene expression level), which was utilized to separate our samples into two groups (HR and LR group) in both the TCGA training cohort and GEO testing cohort.

### Functional enrichment analysis

In this work, we first performed gene differential analysis of the HR group and LR group and identified 10407 and 1922 significantly different genes in the TCGA dataset and GEO dataset, respectively. These genes were used in the Database for Annotation, Visualization and Integrated Discovery (DAVID) (https://david.ncifcrf.gov/) to run gene ontology (GO) and Kyoto Encyclopedia of Genes and Genomes (KEGG) enrichment analyses for the two risk groups. For gene set enrichment analysis (GSEA), TCGA and GEO transcriptome data and risk files were input into R software. The program performs enrichment analysis by sorting the expression profile data, calculating enrichment scores, estimating significance levels, and performing multiple hypothesis testing.

### Statistical analysis

We employed one-way ANOVA and t tests to compare two risk groups, and comparisons of two or more component percent rates were made using the chi-square test. In addition, heatmaps, forest graphs, box plots and receiver operating characteristic (ROC) curves were completed by R software (version 3.5.1). Waterfall curves were completed by MAF tools and R software. Otherwise, Kaplan-Meier analysis was performed to estimate the survival and biochemical recurrence of PCa patients in both the TCGA cluster and two risk groups in the model. We processed all statistical analyses by utilizing SPSS 19.0 software (SPSS. Inc., Chicago, IL, USA) or R software. In our study, a P-value < 0.05 was statistically significant.

### Data availability statement

All data in this study are available in the TCGA data portal (https://tcga-data.nci.nih.gov/tcga/) and GEO data portal (http://www.ncbi.nlm.nih.gov/geo/).

## Supplementary Material

Supplementary Figures

Supplementary Table 1

Supplementary Table 2

Supplementary Table 3

Supplementary Table 4
