# Supplementary material for: Comprehensive analysis of integrin αvβ3/α6β1 in prognosis and immune escape of prostate cancer
Source: Aging (Albany NY). 2023 Oct 19;15(20):11369–88. doi: 10.18632/aging.205131 (PMC10637796; doi:10.18632/aging.205131)
Supplement: Supplementary Figures [file aging-15-205131-s001.pdf]

SUPPLEMENTARY FIGURES

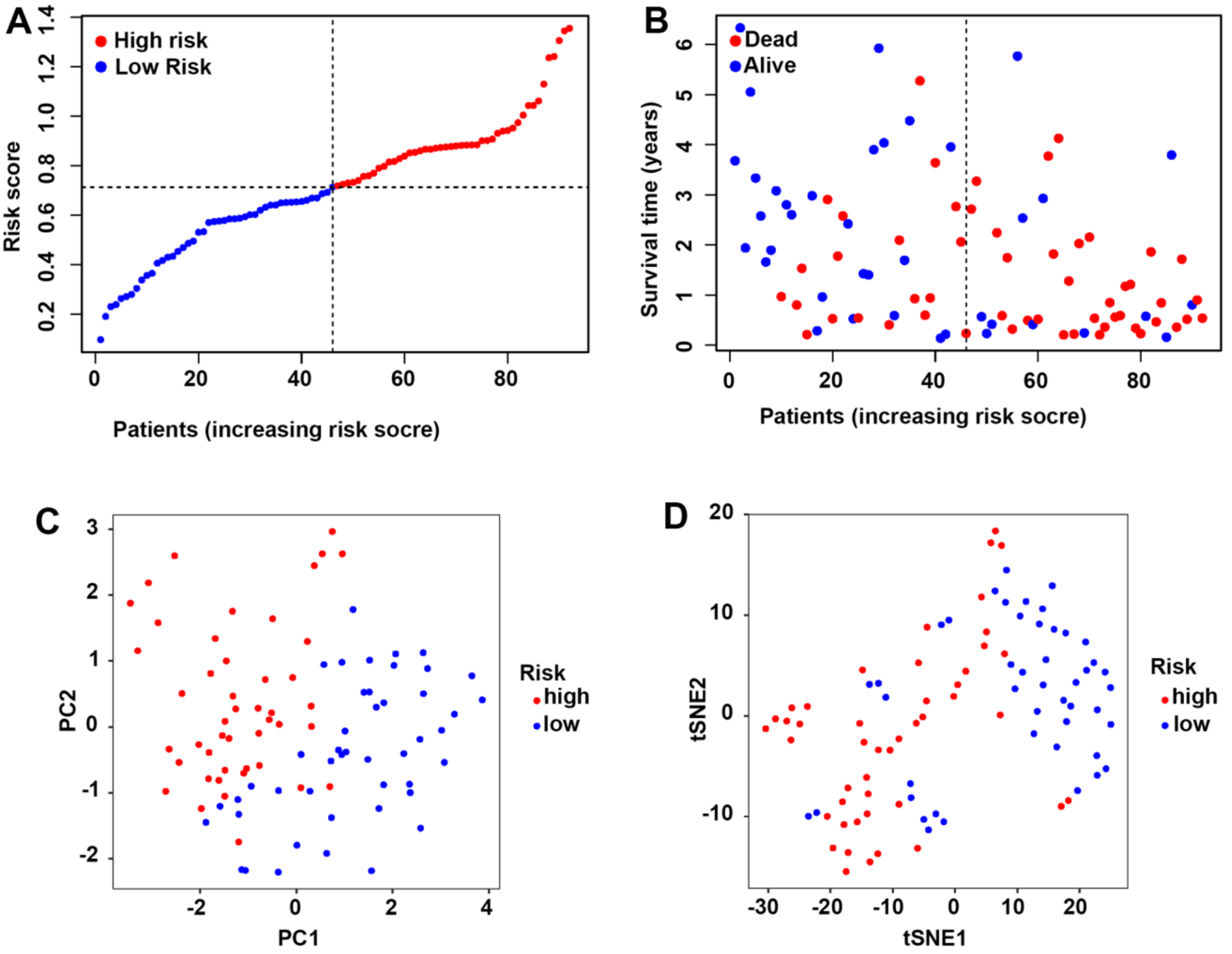

Supplementary Figure 1. (**A**) Ninety-two PCa patients with biochemical recurrence were separated into the HR group and LR group based on the median risk score in the TCGA cohort. (**B**) Survival status of 92 PCa patients with biochemical recurrence in the TCGA cohort. (**C**, **D**) Principal component analysis (PCA) and t-distributed stochastic neighbor embedding (t-SNE) plot for the two risk groups in the TCGA cohort.

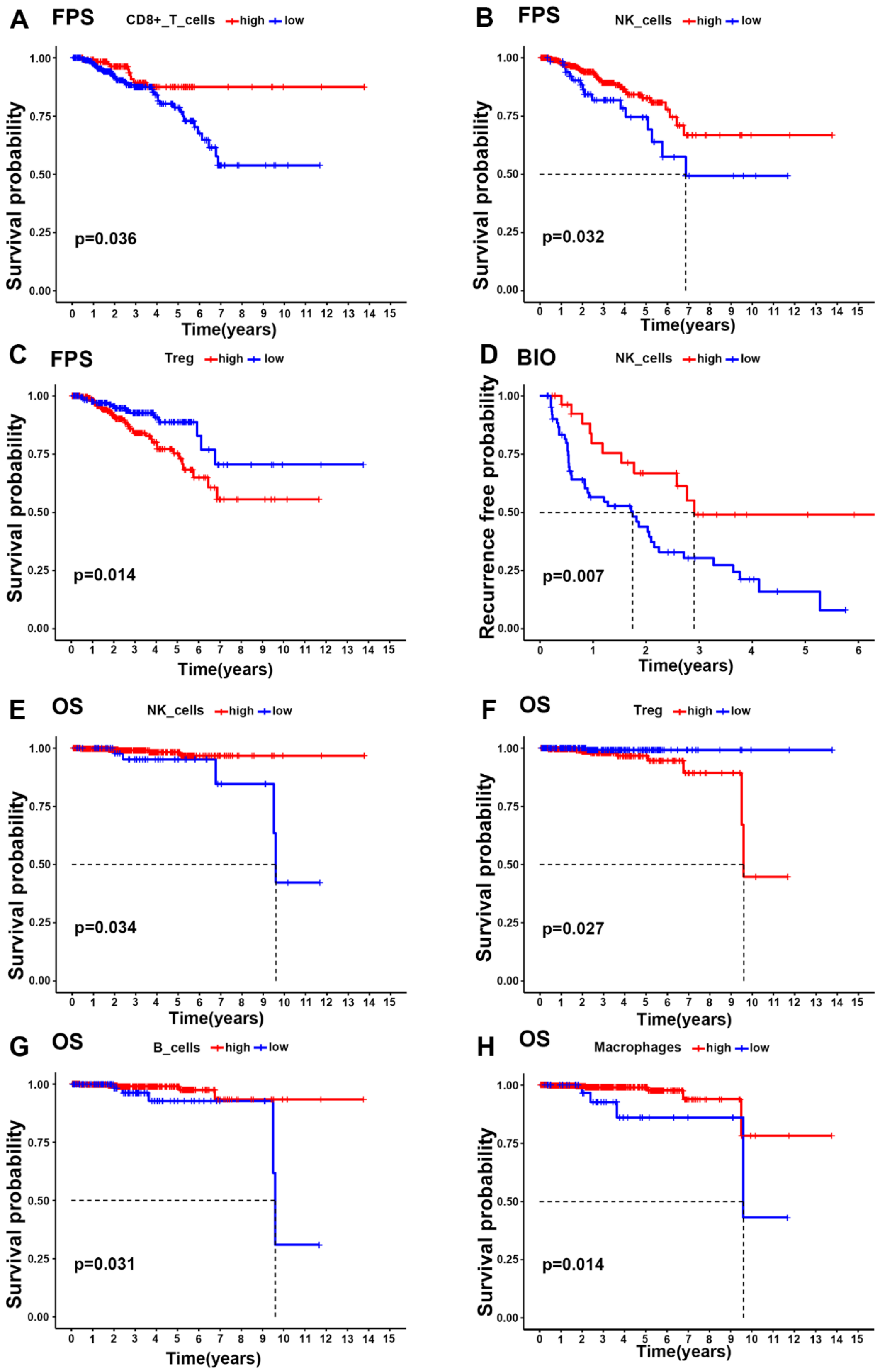

Supplementary Figure 2. (**A**–**C**) Kaplan–Meier analysis for PFS of $CD8^+$ T cells (**A**), NK cells (**B**) and Treg cells (**C**) in two risk groups in the TCGA cohort. (**D**) Kaplan–Meier analysis of the biochemical recurrence of NK cells in the two risk groups in the TCGA cohort. (**E**–**H**) Kaplan–Meier analysis for OS of NK cells (**E**), Treg cells (**F**), B cells (**G**) and macrophages (**H**) in two risk groups in the TCGA cohort.

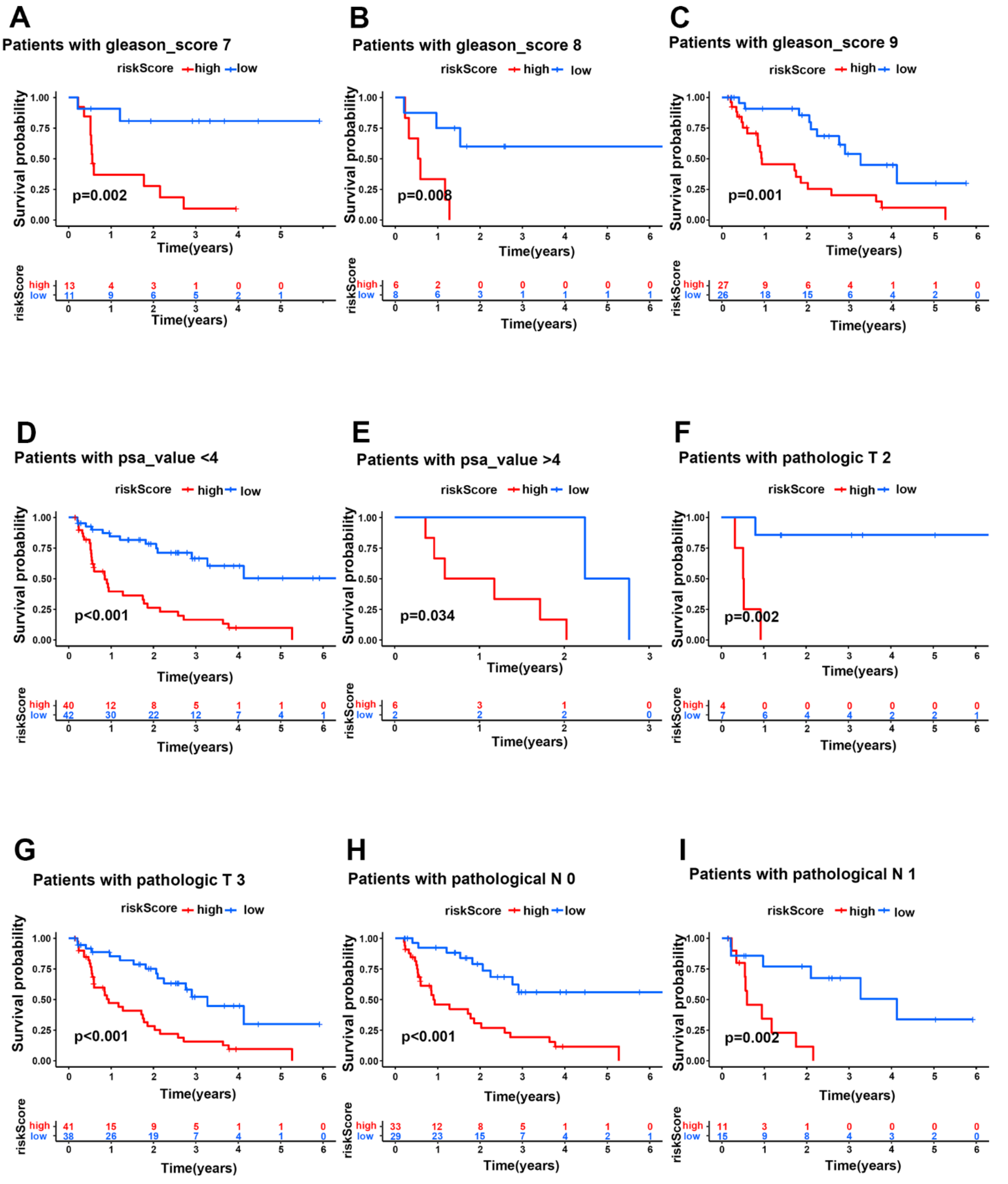

Supplementary Figure 3. (**A**–**I**) Kaplan–Meier survival analysis for PCa patients under different subgroups of clinical characteristics including Gleason score 7-9 (**A**–**C**), PSA-value >4 and PSA-value <4 (**D**, **E**), pathologic T2, T3, N0 and N1 (**F**–**I**) in two risk groups in TCGA cohort.
